# Factors influencing healthcare workers’ attitudes toward delayed retirement: a cross-sectional survey

**DOI:** 10.1186/s12889-025-24449-7

**Published:** 2025-09-24

**Authors:** Xingyu Sun, Zaichun Wu, Lijuan He, Shaohua Wang

**Affiliations:** 1 Department of Gynecology, The Affiliated Traditional Chinese Medicine Hospital, Southwest Medical University, Luzhou, Sichuan 646000 China; 2 Department of Health Management Center, The Affiliated Hospital, Southwest Medical University, Luzhou, Sichuan China; 3 Department of Pathology, The Affiliated Hospital, Southwest Medical University, 25 Taiping Street, Luzhou, China

**Keywords:** Delayed retirement, Healthcare workers, Job satisfaction, Occupational fatigue, Workforce planning

## Abstract

**Background:**

Amid growing concerns over healthcare workforce shortages in aging societies, delayed retirement has emerged as a strategic policy response. However, little is known about the determinants of retirement attitudes across different healthcare professions in middle-income countries.

**Objectives:**

Guided by Role Theory and the Push-Pull Model, this study aimed to identify demographic, occupational, and psychosocial predictors of support for delayed retirement among healthcare workers in China, with attention to inter-professional variation.

**Methods:**

A cross-sectional survey was conducted among 1,200 full-time healthcare workers in Sichuan Province, including doctors, nurses, technicians, and administrative staff. A structured questionnaire captured data on demographics, work conditions, job satisfaction, occupational fatigue, self-rated health, and chronic illness. Univariate and multivariate logistic regression analyses were used to identify independent predictors of support for delayed retirement.

**Results:**

Support for delayed retirement was positively associated with older age (OR: 1.06), male gender (OR: 1.34), higher education (OR: 1.42–1.65), longer working hours, more frequent night shifts, and higher job satisfaction (OR: 1.55), while greater occupational fatigue was negatively associated (OR: 0.82; all *p* < 0.01). Supporters reported better health, lower fatigue, and greater career engagement. Subgroup comparisons revealed marked differences in predictors and attitudes across professional roles, reflecting distinct role identities and workplace demands.

**Conclusions:**

By applying retirement theory to a diverse healthcare sample, this study highlights the need for differentiated workforce retention strategies. Findings suggest that policies should account for occupational fatigue, gendered caregiving burdens, and role-based professional motivations to ensure sustainable retirement planning in resource-constrained health systems.

**Supplementary information:**

The online version contains supplementary material available at 10.1186/s12889-025-24449-7.

## Introduction

As global populations age, governments are under mounting pressure to reform retirement policies to maintain economic productivity and reduce the fiscal burden on public pension systems. Among all sectors, healthcare is particularly vulnerable to these demographic changes, as it depends heavily on a skilled and experienced workforce while simultaneously facing increasing demand for services driven by aging populations. Recent studies highlight that healthcare systems worldwide are experiencing critical shortages of medical professionals, exacerbated by rising care needs and insufficient workforce replenishment [1, 2]. In response to mounting workforce shortages and the increasing demand for continuous healthcare delivery, delayed retirement has been recognized as a strategic policy intervention in health workforce planning. Recent studies have shown that extending the working life of experienced healthcare professionals contributes significantly to alleviating staff shortages and sustaining care continuity, particularly in aging healthcare systems [3, 4].

The decision to retire is multifaceted, involving personal, occupational, economic, and social considerations. Several theoretical frameworks have been proposed to understand retirement behavior. This study draws specifically on Role Theory and the Push-Pull Model of Retirement, which jointly inform our conceptual lens and provide a multi-dimensional understanding of healthcare workers’ retirement intentions. Role Theory emphasizes the psychological and social functions of occupational roles. It posits that professional identity provides structure, purpose, and interpersonal engagement; thus, retirement—especially among professionals in caregiving roles like physicians and nurses—may be experienced as a loss of meaning or social role discontinuity [5]. In contrast, the Push-Pull Model offers a behavioral and motivational perspective, framing retirement as the result of competing pressures: push factors (e.g., fatigue, poor health, job dissatisfaction) drive exit, while pull factors (e.g., job satisfaction, institutional support, and financial need) promote continued labor force participation [6, 7].

These two frameworks are conceptually distinct yet complementary: Role Theory addresses why individuals may be psychologically motivated to continue working, while the Push-Pull Model identifies what conditions lead to early or extended retirement. Integrating them allows for a more holistic assessment of both internal role-related motivations and external environmental forces. This integration is particularly valuable in healthcare settings, where professional identity is strong and work-related strain is high.

**Fig. 1:**
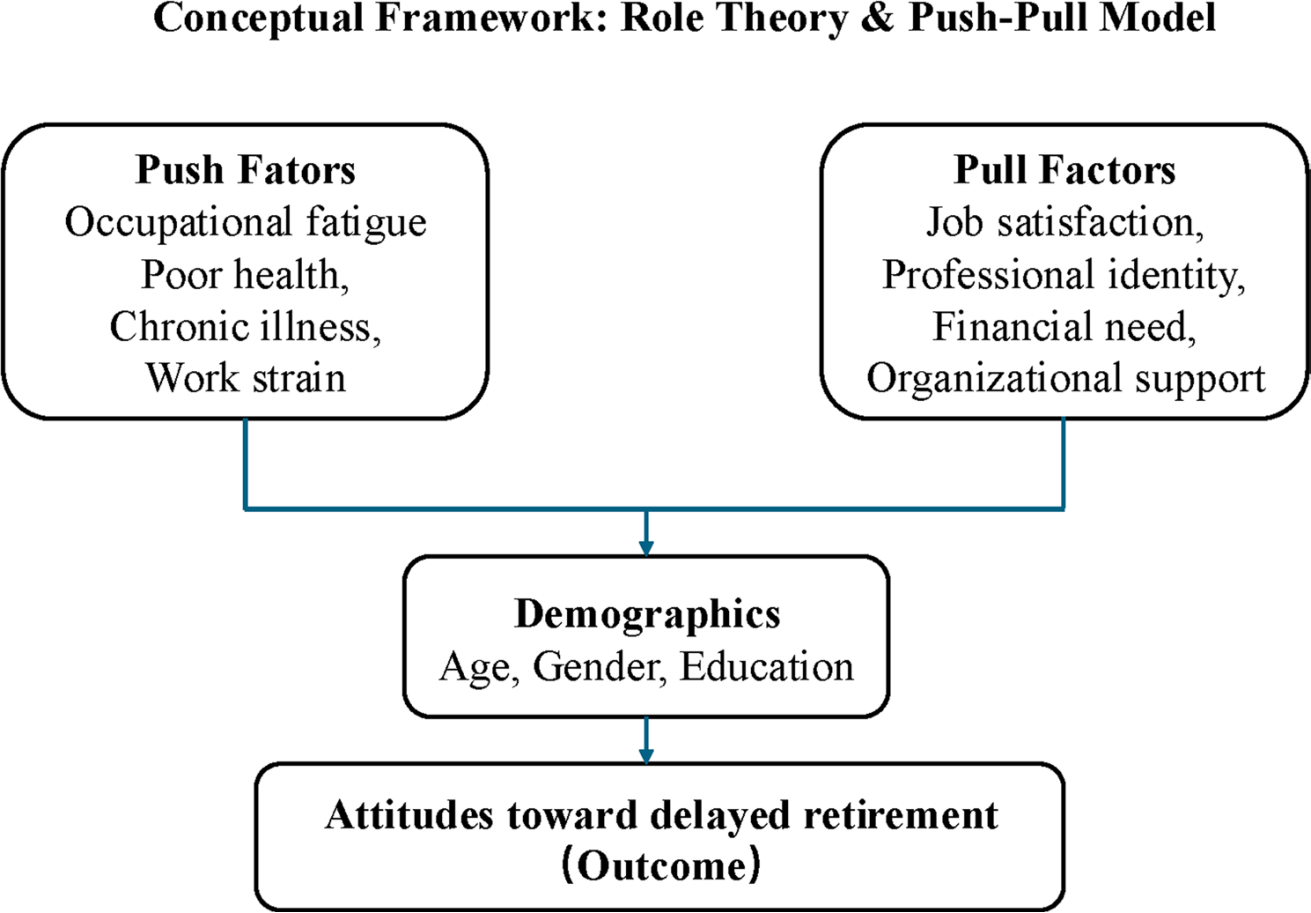
Conceptual framework based on role theory and push-pull model guiding healthcare workers’ attitudes toward delayed retirement

Accordingly, our questionnaire and analytic model were designed to operationalize these theoretical constructs. Variables such as occupational fatigue and self-rated health were conceptualized as push factors, while job satisfaction, educational attainment, and weekly working hours were treated as pull or role-sustaining elements. Demographic factors (e.g., age, gender) were included as potential moderators of these theoretical pathways. Analyses were stratified by professional role, in recognition that the psychological meaning of work and exposure to push-pull dynamics may differ across doctors, nurses, technicians, and administrators. These theoretical relationships are depicted in Fig. 1, which served as a guide for both instrument design and statistical modeling.

Effective workforce planning in healthcare necessitates a dual focus: quantitatively forecasting staffing needs and qualitatively addressing factors like occupational wellbeing, job satisfaction, and burnout risk. Recent studies highlight that neglecting these qualitative aspects can lead to increased staff turnover and compromised patient care quality [8, 9]. Healthcare professionals often face high physical and emotional demands, irregular hours, and exposure to high-stress environments. These factors contribute to occupational fatigue, a significant determinant of early retirement intentions and workforce attrition [10, 11]. Conversely, higher job satisfaction, professional identity, and cognitive engagement have been shown to positively influence decisions to prolong work life [12, 13].

Existing research on retirement decisions has largely focused on the general population or specific high-income settings [14]. However, comparatively fewer studies have examined the complex interplay of demographic, occupational, and health-related factors affecting retirement intentions among healthcare workers in low- and middle-income countries, where systemic constraints and workforce pressures may differ considerably [15, 16]. Moreover, prior studies often overlook the heterogeneity of professional roles within healthcare systems (e.g., doctors vs. nurses vs. technicians), which may shape retirement attitudes differently due to variations in job demands, career trajectories, and work environments [17, 18].

Therefore, this study seeks to fill a critical gap by offering a theoretically informed, profession-specific analysis of the factors influencing healthcare workers’ attitudes toward delayed retirement. By integrating Role Theory and the Push-Pull Model into our conceptual framework, and applying these perspectives to a real-world sample of Chinese healthcare workers, we aim to generate actionable insights for workforce retention policy amid ongoing demographic transitions. In March 2023, the Chinese government formally announced a phased plan to gradually increase the statutory retirement age starting in January 2025, marking a pivotal shift in national labor and pension policy. Healthcare workers are expected to be among the first professional groups affected due to persistent shortages and their critical role in population health. Although this study was conducted prior to the official policy rollout, it coincided with a period of active policy debate, growing public attention, and institutional anticipation. As such, our investigation captures a timely baseline understanding of healthcare workers’ attitudes toward delayed retirement—prior to the confounding influence of formal mandates or implementation strategies.The research question that guides this study is as follows: What demographic, occupational, and psychosocial factors are associated with support for delayed retirement among Chinese healthcare workers, and how do these factors vary across professional subgroups? Addressing this question is essential for anticipating differential responses to retirement reform, informing retention strategies, and mitigating potential resistance among specific occupational groups. By providing a theory-driven, profession-specific analysis from a middle-income healthcare context, our study offers empirical evidence to inform the practical implementation of China’s delayed retirement policy. The findings may also serve as a reference for other countries undergoing similar demographic transitions and workforce aging challenges.

To reflect the integrated nature of workforce planning in Sichuan’s public hospitals, this study included four major occupational groups: physicians, nurses, technicians, and administrative staff. While physicians and nurses deliver direct clinical care and often face higher physical and emotional demands, technicians and administrative staff play essential roles in diagnostics, logistics, and institutional management. In China’s provincial-level human resource planning, particularly under the centralized management of public hospitals, staffing and retirement policies are typically formulated collectively across all categories of healthcare personnel. Thus, analyzing these groups together enhances policy relevance and reflects the way retirement reform is expected to be implemented in practice.

Furthermore, Sichuan Province—located in western China—is experiencing healthcare workforce transitions characterized by resource constraints, an aging labor force, and uneven talent distribution. A comprehensive analysis of multiple occupational subgroups provides a more holistic assessment of workforce sustainability and aligns with regional initiatives aimed at retaining both frontline and support staff. Nevertheless, we recognize the significant heterogeneity across professional roles. To address this, our analysis includes subgroup comparisons to uncover role-specific trends and contextual needs, thereby enhancing the applicability of our findings to occupationally tailored policy interventions.

## Methods

### Study design

This study adopted a cross-sectional survey design to examine the demographic, occupational, and psychosocial factors associated with healthcare workers’ attitudes toward delayed retirement. The study was conducted from January 2023 to January 2024 in Sichuan Province, China. A structured self-administered questionnaire was distributed in both paper and electronic formats to participants from four major occupational groups.

### Sample size estimation

The required sample size was estimated based on established recommendations for logistic regression analysis, which emphasize the importance of having an adequate number of outcome events per predictor variable to maintain model reliability. Assuming a conservative requirement of 10–15 events per variable and planning for 12 to 15 independent variables, we calculated that a minimum of approximately 450 participants would be needed if the outcome proportion (i.e., support for delayed retirement) was around 50%. To improve subgroup representativeness across the four occupational groups and ensure sufficient power to detect small-to-moderate effect sizes (OR = 1.4–1.6) at α = 0.05, we targeted a total sample size of 1,200 participants. All valid responses were included in the final analysis.

### Survey instrument, pretesting, and validity assessment

Data were collected using a self-administered questionnaire specifically developed for this study, available in both electronic and paper formats. The instrument consisted of six sections (see Supplementary File 1): (1) demographic characteristics; (2) work-related factors; (3) job satisfaction; (4) occupational fatigue; (5) self-rated health and chronic illness; and (6) attitudes toward delayed retirement.

Job satisfaction was assessed using a single-item 5-point Likert scale (1 = Very dissatisfied to 5 = Very satisfied), adapted from the short-form Minnesota Satisfaction Questionnaire, originally developed by Weiss et al. [19]. This item has been widely used in occupational research, including among healthcare workers, and demonstrated acceptable internal consistency in our pretest sample (Cronbach’s α = 0.82).

Occupational fatigue was measured using a 10-point single-item scale ranging from 1 (no fatigue) to 10 (extreme fatigue), based on the Occupational Fatigue Exhaustion Recovery (OFER) model proposed by Winwood et al. [20]. This simplified version showed good reliability in our pretest (Cronbach’s α = 0.85) and was well accepted by participants.

Self-rated health was measured using a commonly applied single-item general health question: “How would you rate your overall health?”, with response options ranging from “excellent” to “poor,” following the format used by DeSalvo et al. [21]. Chronic illness was assessed with a binary item asking whether the respondent had been diagnosed with any chronic condition (e.g., hypertension, diabetes), with an optional follow-up to specify diagnoses. As these items are single-item or dichotomous indicators, Cronbach’s alpha is not applicable; however, both measures are widely validated and have demonstrated robust predictive validity in prior epidemiological studies.

Attitudes toward delayed retirement, the primary outcome of interest, were assessed using two items: (1) a 5-point Likert item (“How likely are you to support delayed retirement?”), and (2) a multiple-choice question indicating preferred retirement age. While we acknowledge the conceptual limitations of using only two items, these were developed with reference to retirement decision literature (e.g., Shultz et al. [5]; AlKhars et al. [6]) and tailored to our study context. Due to the lack of an existing validated scale for delayed retirement attitudes in Chinese healthcare populations, our design emphasized brevity and clarity to enhance response rates and minimize respondent burden in a clinical setting.

To assess the content validity of the questionnaire, a panel of five experts—including senior healthcare administrators, occupational psychologists, and public health researchers—independently reviewed the initial draft for item relevance, clarity, and theoretical alignment. Based on their feedback, minor modifications were made to improve face and content validity. For construct validity, we performed an exploratory factor analysis (EFA) using principal axis factoring with oblique rotation on the four attitudinal and psychological items: job satisfaction, occupational fatigue, support for delayed retirement, and preferred retirement age. The Kaiser-Meyer-Olkin (KMO) measure of sampling adequacy was 0.74 and Bartlett’s test of sphericity was statistically significant (χ² = 268.3, df = 6, *p* < 0.001), supporting factorability. A one-factor solution explained 58.4% of the total variance, consistent with the underlying theoretical construct of “retirement intention climate.” Furthermore, all applicable single-item constructs demonstrated good convergent validity, with moderate to strong inter-item correlations (*r* = 0.43–0.62, *p* < 0.001), particularly between job satisfaction and support for delayed retirement. These findings indicate that the instrument possesses adequate psychometric properties for exploratory research in time-constrained clinical settings.

The full English version of the questionnaire is provided in Supplementary File 1. Pretesting with 30 healthcare workers confirmed the instrument’s face validity, acceptability, and item clarity. Minor wording adjustments were made based on their feedback. The overall internal consistency across applicable domains (i.e., job satisfaction and fatigue) was acceptable (combined Cronbach’s α = 0.84). The decision to use concise, single-item measures was guided by practical considerations of feasibility in real-world healthcare environments, where time constraints and operational demands may limit participation in lengthy surveys.

The development of the questionnaire was guided by both Role Theory and the Push-Pull Model of retirement. Specifically, Role Theory informed the inclusion of variables that reflect professional identity, role continuity, and job satisfaction, particularly relevant to doctors and nurses who may derive strong personal meaning from their clinical roles. For example, the single-item measure of job satisfaction was included to assess the extent to which individuals find their work fulfilling, which aligns with the theory’s emphasis on role-based engagement and continuity as incentives for delayed retirement. The Push-Pull Model shaped the selection of variables categorized as either facilitating or deterring extended workforce participation. Occupational fatigue and self-rated health were conceptualized as “push” factors—conditions that may compel individuals toward early retirement due to physical or psychological strain. Conversely, higher education level, longer working hours, and greater job satisfaction were included as “pull” factors, potentially encouraging prolonged employment due to professional engagement or institutional expectations. The two-item measure of attitudes toward delayed retirement was likewise derived from this dual-framework lens: the Likert-style item captured attitudinal openness to policy change (conceptually linked to role continuity and job satisfaction), while the preferred retirement age question reflected behavioral intention shaped by both “push” and “pull” influences. This theoretically driven approach enabled a more coherent analytical structure, where observed associations could be interpreted within a validated conceptual framework.

### Data collection

Data were collected from January 2023 to January 2024 using a structured, self-administered questionnaire developed specifically for this study. The questionnaire was made available in both electronic and paper formats to accommodate participants’ preferences. It was designed to gather information on demographic characteristics (e.g., age, gender, education level, and marital status), work-related factors (e.g., work experience, weekly working hours, and night shift frequency), and health status (e.g., self-rated health and the presence of chronic illness). Additionally, the questionnaire assessed key variables such as job satisfaction, occupational fatigue, and attitudes toward delayed retirement. An English version of the questionnaire is provided as a supplementary file (see Supplementary File 1). Paper questionnaires were distributed in person during departmental meetings by trained research assistants, who also collected completed forms in sealed envelopes to ensure confidentiality. Electronic questionnaires were disseminated via institutional email and workplace WeChat groups using a secure survey platform with one-time access links.

### Statistical analysis

Descriptive statistics were used to summarize the demographic and work-related characteristics of the sample. Continuous variables were presented as means and standard deviations (SD), while categorical variables were reported as frequencies and percentages.

To assess factors associated with attitudes toward delayed retirement, both univariate and multivariate logistic regression analyses were performed. In the univariate analysis, odds ratios (OR) and 95% confidence intervals (CI) were calculated to identify potential predictors of support for delayed retirement. Variables with a p-value < 0.05 in the univariate analysis were included in the multivariate logistic regression model to account for potential confounding factors.

Multivariate logistic regression analysis was used to identify independent predictors of support for delayed retirement. Adjusted ORs and 95% CIs were reported for all variables included in the final model. Statistical significance was set at *p* < 0.05 for all analyses. All statistical analyses were conducted using SPSS version 26.0 (IBM Corp., Armonk, NY).

## Results

### Demographic and occupational characteristics of healthcare workers

Table 1 summarizes the characteristics of 1,200 healthcare workers stratified by profession. Significant group differences were observed across all variables (*p* < 0.05). Doctors and administrative staff were the oldest (mean ages: 47.2 and 48.5 years, respectively), while nurses were the youngest (43.8 years). Gender distribution varied markedly: 75.0% of doctors were male, whereas 89.6% of nurses were female. Education level also differed significantly; 75.0% of doctors held a master’s degree or higher, while 64.0% of nurses had a bachelor’s degree. Marital status showed that 74.8% of respondents were married, with the highest proportion among doctors (82.0%). Doctors also had the longest work experience (23.5 years), the longest weekly working hours (50.2 h), and the highest night shift frequency (6.5 per month). In contrast, administrative staff had the fewest night shifts (2.1 per month) and lowest proportion reporting excellent/good health (50.0%). Self-rated health and chronic illness differed across groups. Poor health was most common among nurses (13.6%), while the prevalence of chronic illness was highest among technicians (43.0%) and administrative staff (40.0%) (*p* = 0.045). These patterns highlight the heterogeneity of healthcare worker subgroups, potentially shaping their retirement-related preferences.

**Table 1:** Demographic characteristics of healthcare workers with group comparisons (*n* = 1200)

Variable	Overall (*n* = 1200)	Doctors (*n* = 400)	Nurses (*n* = 500)	Technicians (*n* = 200)	Admin Staff (*n* = 100)	*p*-value
Age (years), Mean (SD)	45.6 (8.2)	47.2 (7.9)	43.8 (8.1)	46.1 (7.5)	48.5 (6.9)	< 0.001**
Gender, n (%)						< 0.001**
- Male	502 (41.8%)	300 (75.0%)	52 (10.4%)	100 (50.0%)	50 (50.0%)	
- Female	698 (58.2%)	100 (25.0%)	448 (89.6%)	100 (50.0%)	50 (50.0%)	
Education Level, n (%)						< 0.001**
- High School or below	135 (11.3%)	10 (2.5%)	80 (16.0%)	30 (15.0%)	15 (15.0%)	
- Bachelor’s Degree	515 (42.9%)	90 (22.5%)	320 (64.0%)	80 (40.0%)	25 (25.0%)	
- Master’s Degree or Higher	550 (45.8%)	300 (75.0%)	100 (20.0%)	90 (45.0%)	60 (60.0%)	
Marital Status, n (%)						0.043*
- Married	898 (74.8%)	328 (82.0%)	340 (68.0%)	160 (80.0%)	70 (70.0%)	
- Single	204 (17.0%)	52 (13.0%)	100 (20.0%)	22 (11.0%)	30 (30.0%)	
- Divorced/Widowed	98 (8.2%)	20 (5.0%)	60 (12.0%)	18 (9.0%)	0 (0.0%)	
Work Experience (years), Mean (SD)	20.4 (9.6)	23.5 (8.2)	18.2 (9.1)	19.6 (8.4)	22.1 (7.5)	< 0.001**
Weekly Working Hours, Mean (SD)	46.5 (8.9)	50.2 (7.8)	42.8 (8.7)	45.5 (8.0)	40.2 (7.3)	< 0.001**
Night Shift Frequency/month, Mean (SD)	4.6 (3.2)	6.5 (3.5)	3.8 (2.7)	4.1 (3.0)	2.1 (1.5)	< 0.001**
Self-Rated Health, n (%)						0.018*
- Excellent/Good	789 (65.8%)	310 (77.5%)	312 (62.4%)	117 (58.5%)	50 (50.0%)	
- Fair	312 (26.0%)	72 (18.0%)	120 (24.0%)	60 (30.0%)	60 (60.0%)	
- Poor	99 (8.2%)	18 (4.5%)	68 (13.6%)	23 (11.5%)	0 (0.0%)	
Chronic Illness, n (%)						0.045*
- Yes	454 (37.8%)	148 (37.0%)	180 (36.0%)	86 (43.0%)	40 (40.0%)	
- No	746 (62.2%)	252 (63.0%)	320 (64.0%)	114 (57.0%)	60 (60.0%)	

Values are presented as mean (SD) for continuous variables and n (%) for categorical variables. *p*-values are derived from ANOVA for continuous variables and chi-square test for categorical variables. *p* < 0.05; *p* < 0.001

### Univariate and multivariate analysis of factors associated with support for delayed retirement

Table 2 shows that in both univariate and multivariate logistic regression analyses, older age, male gender, higher education level, longer work experience, greater weekly working hours, and more frequent night shifts were significantly associated with support for delayed retirement (all *p* < 0.05). Job-related factors demonstrated particularly strong associations: each additional work hour per week (OR: 1.06, 95% CI: 1.03–1.10) and each additional night shift per month (OR: 1.09, 95% CI: 1.04–1.14) increased the likelihood of support. Compared to high school education, holding a bachelor’s degree (OR: 1.35) or master’s degree or above (OR: 1.55) was also positively associated. While self-rated health and chronic illness were significant in univariate models, they were not retained in the final multivariate model, suggesting potential mediation by other variables.

**Table 2:** Univariate and multivariate analysis of factors associated with attitudes toward delayed retirement among healthcare workers (*n* = 1200)

Variable	Univariate Analysis (OR, 95% CI)	*p*-value	Multivariate Analysis (OR, 95% CI)	*p*-value
Age (years)	1.05 (1.03–1.07)	< 0.001**	1.04 (1.02–1.06)	< 0.001**
Gender (Male)	1.30 (1.10–1.54)	0.002**	1.25 (1.03–1.51)	0.021*
Education Level				
- High School	Reference		Reference	
- Bachelor’s Degree	1.45 (1.20–1.75)	< 0.001**	1.35 (1.08–1.68)	0.007**
- Master’s or Higher	1.78 (1.40–2.26)	< 0.001**	1.55 (1.18–2.04)	0.002**
Work Experience (yrs)	1.02 (1.01–1.04)	0.008**	1.01 (1.00–1.03)	0.032*
Weekly Working Hours	1.08 (1.05–1.11)	< 0.001**	1.06 (1.03–1.10)	< 0.001**
Night Shift Frequency	1.12 (1.08–1.16)	< 0.001**	1.09 (1.04–1.14)	< 0.001**
Self-rated Health				
- Excellent/Good	Reference		Reference	
- Fair	1.23 (1.01–1.50)	0.042*	1.15 (0.94–1.41)	0.158
- Poor	1.48 (1.10–2.00)	0.009**	1.28 (0.92–1.78)	0.137
Chronic Illness (Yes)	1.22 (1.03–1.45)	0.024*	1.14 (0.94–1.39)	0.180

Odds ratios (OR) and 95% confidence intervals (CI) are shown for both univariate and multivariate logistic regression analyses. Variables with *p*-values less than 0.05 were considered statistically significant. Asterisks indicate significance levels: *p* < 0.05; *p* < 0.001

### Comparison of psychosocial and health characteristics by attitudes toward delayed retirement

As shown in Table 3, healthcare workers who supported delayed retirement reported higher job satisfaction (mean = 4.3 vs. 3.8, *p* < 0.001) and lower occupational fatigue (mean = 6.1 vs. 7.0, *p* < 0.001) than those who opposed it. Supporters also reported better self-rated health, with a higher proportion rating their health as excellent or good (72.0% vs. 57.8%, *p* < 0.001), and fewer reporting poor health (5.3% vs. 13.3%). Chronic illness prevalence was higher among those who opposed delayed retirement, with 46.7% reporting chronic illness compared to 33.3% of supporters (*p* = 0.002). In terms of work conditions, supporters reported longer weekly working hours (48.3 vs. 43.6, *p* < 0.001) and more frequent night shifts (5.2 vs. 3.7 per month, *p* < 0.001).

**Table 3:** Comparison of job satisfaction, occupational fatigue, and health status by attitudes toward delayed retirement among healthcare workers (*n* = 1200)

Variable	Support Delayed Retirement (*n* = 750)	Oppose Delayed Retirement (*n* = 450)	*p*-value
Job Satisfaction, Mean (SD)	4.3 (0.8)	3.8 (1.0)	< 0.001**
Occupational Fatigue, Mean (SD)	6.1 (1.2)	7.0 (1.5)	< 0.001**
Self-rated Health, n (%)			< 0.001**
- Excellent/Good	540 (72.0%)	260 (57.8%)	
- Fair	170 (22.7%)	130 (28.9%)	
- Poor	40 (5.3%)	60 (13.3%)	
Chronic Illness, n (%)			0.002**
- Yes	250 (33.3%)	210 (46.7%)	
- No	500 (66.7%)	240 (53.3%)	
Weekly Working Hours, Mean (SD)	48.3 (8.5)	43.6 (8.3)	< 0.001**
Night Shift Frequency (per month), Mean (SD)	5.2 (3.1)	3.7 (2.9)	< 0.001**

Data are presented as means (SD) for continuous variables and counts (percentages) for categorical variables. *p*-values were calculated using t-tests for continuous variables and chi-square tests for categorical variables. Asterisks indicate significance at *p* < 0.001

### Multivariate logistic regression analysis of factors associated with support for delayed retirement

As shown in Table 4, age (OR = 1.06, 95% CI: 1.04–1.08, *p* < 0.001) and male gender (OR = 1.34, 95% CI: 1.12–1.61, *p* = 0.002) were significant positive predictors of support for delayed retirement. Compared to those with a high school education, healthcare workers with a bachelor’s degree (OR = 1.42, 95% CI: 1.12–1.80, *p* = 0.003) or a master’s degree or higher (OR = 1.65, 95% CI: 1.21–2.24, *p* = 0.001) were more likely to support delayed retirement. Greater work experience (OR = 1.02, 95% CI: 1.00–1.04, *p* = 0.045), longer weekly working hours (OR = 1.07, 95% CI: 1.05–1.10, *p* < 0.001), and higher night shift frequency (OR = 1.10, 95% CI: 1.06–1.14, *p* < 0.001) were also positively associated with support. Job satisfaction was strongly associated with support (OR = 1.55, 95% CI: 1.30–1.85, *p* < 0.001), while occupational fatigue was inversely associated (OR = 0.82, 95% CI: 0.74–0.91, *p* < 0.001). Self-rated health and chronic illness were not statistically significant predictors in the multivariate model.

**Table 4:** Multivariate logistic regression analysis of factors associated with support for delayed retirement among healthcare workers (*n* = 1200)

Variable	OR (95% CI)	*p*-value
Age (years)	1.06 (1.04–1.08)	< 0.001**
Gender (Male)	1.34 (1.12–1.61)	0.002**
Education Level		
- High School	Reference	
- Bachelor’s Degree	1.42 (1.12–1.80)	0.003**
- Master’s or Higher	1.65 (1.21–2.24)	0.001**
Work Experience (yrs)	1.02 (1.00–1.04)	0.045*
Weekly Working Hours	1.07 (1.05–1.10)	< 0.001**
Night Shift Frequency	1.10 (1.06–1.14)	< 0.001**
Job Satisfaction	1.55 (1.30–1.85)	< 0.001**
Occupational Fatigue	0.82 (0.74–0.91)	< 0.001**
Self-rated Health		
- Excellent/Good	Reference	
- Fair	1.20 (0.97–1.49)	0.091
- Poor	1.38 (0.98–1.95)	0.065
Chronic Illness (Yes)	0.89 (0.72–1.11)	0.305

Odds ratios (OR) and 95% confidence intervals (CI) are shown for multivariate logistic regression analysis. Variables with *p*-values less than 0.05 were considered statistically significant. Asterisks indicate significance at *p* < 0.001; *p* < 0.05

## Discussion

This study aimed to identify demographic, occupational, and psychosocial factors that influence healthcare workers’ attitudes toward delayed retirement. The findings demonstrate that age, gender, education level, job satisfaction, occupational fatigue, and work conditions are significantly associated with support for delaying retirement. These results are conceptually grounded in Role Theory and the Push-Pull Model of Retirement. Specifically, Role Theory suggests that healthcare professionals who strongly identify with their occupational roles—such as physicians or administrators—are more likely to support delayed retirement due to the psychological value and continuity derived from their professional identities. In contrast, the Push-Pull Model explains how external conditions (e.g., high occupational fatigue as a push factor, or high job satisfaction as a pull factor) shape healthcare workers’ willingness to postpone retirement. These theoretical pathways help clarify how individual, occupational, and psychosocial variables causally influence retirement attitudes.

Role Theory suggests that professional identity, meaning, and structure derived from one’s occupation may encourage older healthcare workers, especially those in leadership or academic roles, to postpone retirement. Meanwhile, the Push-Pull Model explains retirement intention as a result of opposing forces: while job satisfaction, institutional support, and financial security act as “pull” factors for continued employment, fatigue, burnout, and health concerns operate as “push” factors prompting early exit [5–7].

Consistent with previous literature, age was one of the most significant predictors. Older healthcare workers are more likely to support delayed retirement, not only because of their proximity to statutory retirement age but also due to the institutional roles they have accrued. Many occupy senior clinical, academic, or administrative positions that come with extensive responsibilities and expectations for mentorship, decision-making, and organizational leadership. These responsibilities often generate a strong sense of professional identity and institutional commitment. According to Role Theory, such occupational continuity provides meaning and social structure, making retirement psychologically and organizationally difficult to initiate. Moreover, evidence suggests that senior professionals often delay retirement to preserve social influence, cognitive engagement, and legacy within their institutions [22–24].

Gender differences were evident: male healthcare workers were more inclined to support delayed retirement compared to female workers. This trend can be explained by both structural and psychosocial factors. From a Push-Pull Model perspective, women are more likely to experience stronger “push” factors such as physical exhaustion from frontline duties, work-family conflict, and dual caregiving roles, especially in female-dominated professions like nursing. These pressures can lead to earlier retirement intentions. Conversely, men may face fewer external caregiving obligations and are more likely to occupy higher-ranking positions with greater job autonomy, financial compensation, and institutional support, which function as “pull” factors toward continued employment. Role Theory also helps explain this gendered pattern. Men often derive status, identity, and self-worth from their professional achievements, especially in leadership or specialist roles. For them, retirement may signify a loss of role-based meaning, leading to stronger psychological motivation to continue working. Prior studies have shown that men in the healthcare sector—particularly physicians—are more likely to extend their careers due to the perceived value of their accumulated expertise and the prestige associated with their roles [25, 26]. In contrast, women may weigh emotional and physical fatigue, family responsibilities, or limited upward mobility more heavily in retirement decisions. These dynamics are evident in our sample, where a higher proportion of male respondents were doctors with postgraduate education and leadership responsibilities (see Table 1), supporting the idea that gender and occupational structure interact to shape retirement attitudes. Therefore, the observed association between male gender and support for delayed retirement likely reflects both differences in role-based motivations and unequal structural conditions across gender lines in the healthcare workforce.

The theoretical framework also helps interpret these observed associations. For example, educational attainment not only reflects socioeconomic status but also indicates role complexity and autonomy, which Role Theory associates with stronger professional identity and resistance to role exit. Similarly, job satisfaction, night shifts, and fatigue can be directly mapped to the motivational forces outlined in the Push-Pull Model—highlighting how these factors influence the central outcome variable of retirement attitude. Education level also played a significant role in shaping attitudes toward delayed retirement. Our findings indicate that healthcare workers with a bachelor’s degree or higher were significantly more likely to support delayed retirement and report higher job satisfaction. This association can be interpreted through both Role Theory and the Push-Pull Model. According to Role Theory, individuals with higher educational attainment often occupy roles with greater autonomy, complexity, and cognitive demands—such as physicians, administrators, or academic staff. These roles provide a strong source of professional identity and meaning, which enhances role continuity and increases the psychological cost of retirement. Higher education also correlates with increased access to leadership positions, greater perceived job control, and higher levels of institutional recognition, all of which reinforce the desire to remain in the workforce. From a Push-Pull perspective, these individuals are more strongly influenced by “pull” factors—such as professional fulfillment, institutional support, and ongoing intellectual engagement—which outweigh “push” factors like fatigue or physical strain that are more common in lower-status or more physically demanding roles. For example, in our sample (Table 1), those with advanced degrees were disproportionately represented among doctors and administrators, both of whom reported higher job satisfaction and longer weekly working hours—indicators of continued career investment. These patterns are consistent with previous research suggesting that individuals in cognitively enriched or high-status professions often delay retirement due to the intrinsic rewards and sense of purpose derived from their work [27, 28]. Thus, the relationship between educational attainment, job satisfaction, and retirement attitudes likely reflects a confluence of role-based psychological motivations, institutional structures, and occupational status, reinforcing the value of considering profession-specific dynamics in retirement policy planning.

In addition to individual-level predictors, notable differences in demographic and occupational characteristics were observed across professional groups, as shown in Table 1. These differences offer further explanatory context for variations in delayed retirement attitudes. For example, doctors were predominantly male (75%), highly educated (75% held a master’s degree or higher), and had the highest weekly working hours (50.2 h) and night shift frequency (6.5 per month). These features align with predictors associated with support for delayed retirement in our multivariate model (Table 4), suggesting that physicians are more strongly influenced by “pull” factors such as job autonomy, institutional responsibility, and professional identity. In contrast, nurses were primarily female (89.6%), younger (mean age: 43.8 years), and had lower educational attainment (only 20% with a master’s degree or above). Despite contributing substantially to frontline care, they reported shorter working hours and higher rates of poor self-rated health (13.6%) and occupational fatigue. These characteristics correspond with stronger “push” factors—such as physical strain, emotional exhaustion, and caregiving burdens—that may discourage delayed retirement. Technicians and administrative staff displayed more heterogeneous profiles. Technicians had moderate work intensity but the highest rate of chronic illness (43.0%), while administrative staff reported the fewest night shifts (2.1 per month) and the lowest proportion of excellent/good health (50.0%), which may influence retirement preferences in both directions depending on their job roles and advancement opportunities. These intergroup variations illustrate how demographic and role-based contexts interact with psychological and institutional forces in shaping retirement intentions. While uniform retirement policies are administratively efficient, our findings suggest that profession-specific considerations are essential for anticipating workforce responses and developing targeted retention strategies. We have expanded the Discussion accordingly to integrate these subgroup-level patterns within our theoretical framework.

Job satisfaction was a robust positive predictor. As expected, those who were more satisfied with their work expressed greater support for delaying retirement. This confirms findings that intrinsic rewards such as professional fulfillment and patient relationships encourage longer service [29]. In contrast, occupational fatigue negatively correlated with support for delayed retirement, consistent with research highlighting burnout and emotional exhaustion as key factors in early workforce departure [30, 31].

Notably, while longer working hours and frequent night shifts were associated with delayed retirement, this may reflect reverse causality: those who continue to work intensively may do so out of necessity or dedication rather than preference [32, 33]. These patterns underscore the importance of improving working conditions and scheduling to accommodate aging workers. While the study adopted a theoretically informed approach grounded in Role Theory and the Push-Pull Model to examine psychosocial and occupational determinants of delayed retirement, certain important dimensions—specifically, perceived work meaning and economic considerations—were not directly measured in this survey. This omission was primarily due to the feasibility constraints of implementing a concise instrument across high-workload clinical settings, where lengthy questionnaires could reduce response rates. Moreover, during our instrument development stage, expert consultations prioritized brevity and clarity to optimize respondent engagement. We acknowledge that the absence of explicit indicators of work meaning (e.g., sense of purpose, professional calling) and financial preparedness (e.g., pension adequacy, income satisfaction) limits a full understanding of motivational dynamics. Future studies may benefit from incorporating validated multi-item tools such as the Work as Meaning Inventory (WAMI) and financial planning scales to capture these constructs more comprehensively. Nonetheless, job satisfaction and educational attainment were included as partial proxies for intrinsic motivation and socioeconomic position, respectively, and align conceptually with role-continuity and pull mechanisms in our framework.

Interestingly, self-rated health and chronic illness were not significant in multivariate models. This may suggest that healthcare workers, due to medical knowledge and access to care, are better able to manage chronic conditions without exiting the workforce, a finding divergent from past studies in other sectors [34, 35].

Our study contributes several novel insights. First, it provides context-specific data from a middle-income country undergoing major retirement policy reform, which has been underrepresented in existing research. Second, it disaggregates findings across four healthcare subgroups—doctors, nurses, technicians, and administrators—allowing for profession-specific implications. Third, by applying Role Theory and the Push-Pull Model to questionnaire design and analysis, we enhance theoretical interpretation of retirement behavior in a clinical context. Lastly, we validate the role of job satisfaction and fatigue using established measures, offering actionable evidence for workforce planning. Furthermore, to address the heterogeneity across professional groups, we conducted subgroup analyses that revealed notable differences in retirement attitudes and influencing factors. Physicians showed relatively higher support for delayed retirement, which may be attributed to their strong professional identity, longer career development trajectories, and greater institutional responsibilities. Many physicians—particularly in senior or academic roles—derive meaning from continued clinical engagement, research, and mentorship, making retirement a more complex and personally consequential decision. Nurses, by contrast, reported lower support for delayed retirement, likely due to the physically and emotionally demanding nature of frontline care, combined with higher rates of occupational fatigue and gendered caregiving responsibilities outside of work. The predominantly female composition of the nursing workforce may further contribute to earlier retirement intentions, as suggested by previous research on work-family conflict and dual caregiving burdens. Technicians and administrative staff exhibited more variable responses. For technicians, factors such as irregular hours, limited upward mobility, and less decision-making autonomy may contribute to moderate levels of retirement intention, influenced more by structural constraints than intrinsic motivation. Administrative personnel, on the other hand, often experience less physical strain but may face career stagnation and lower job satisfaction in non-clinical roles, which could shape their retirement preferences differently. These findings highlight how different professional identities shape retirement attitudes, suggesting that tailored retention policies must account for such occupational nuances and structural differences. While unified retirement policies may simplify implementation, they risk overlooking the diverse needs, capacities, and expectations embedded within different professional identities in healthcare settings.

This study has limitations. Its cross-sectional design precludes causal inference. The sample was drawn from institutions affiliated with a single university in Sichuan Province, limiting generalizability. Although single-item scales were used for job satisfaction and fatigue to improve feasibility, they may lack depth. Moreover, the chronic illness measure did not capture disease type or severity. Future studies should incorporate validated multidimensional tools such as the Maslach Burnout Inventory or Multidimensional Fatigue Inventory to improve psychometric depth.

While this study did not perform separate regression analyses by professional subgroup (e.g., doctors, nurses, technicians, administrators), this decision was guided by both methodological and pragmatic considerations. First, conducting multivariate logistic regression within each subgroup would substantially reduce statistical power, particularly for smaller groups such as technicians (*n* = 200) and administrative staff (*n* = 100). Stratified modeling with limited events per variable could result in unstable estimates and model overfitting, thereby compromising the reliability and generalizability of subgroup-specific findings. Second, the current analysis strategy prioritized identifying robust and generalizable predictors of support for delayed retirement across the full healthcare workforce, in line with national retirement policy planning that typically applies uniform policies across professional groups. That said, we recognize the importance of professional heterogeneity, which is evident in Table 1, where demographic, educational, and work-related characteristics differ significantly across roles. For example, doctors were predominantly male, had the highest levels of education, and worked the longest hours with the most night shifts—all factors positively associated with delayed retirement (Tables 2 and 4). In contrast, nurses—who were younger, predominantly female, and reported lower education levels—are likely influenced more strongly by occupational fatigue and caregiving responsibilities, factors that push toward earlier retirement. Although formal interaction terms or subgroup regressions were not performed, the distinct distribution of key predictors across professional roles indirectly suggests that the determinants of delayed retirement are shaped by role-specific dynamics. We have expanded our Discussion to interpret these observed differences and to highlight the need for future studies to conduct profession-specific modeling with larger stratified samples. Additionally, we have acknowledged the absence of subgroup regression as a study limitation and proposed it as a direction for future research aiming to inform more tailored retention strategies.

This study has several limitations. First, its cross-sectional design precludes causal inference, and findings should be interpreted as associations rather than determinants. Second, although our sample size (*n* = 1,200) was adequate for the overall analyses, we did not perform separate regression models by professional subgroup (e.g., doctors, nurses, technicians, administrators). This decision was made to avoid statistical underpowering and overfitting—particularly for occupational groups such as administrative staff, who naturally represent a smaller proportion of the healthcare workforce in our study settings. The limited number of participants in these subgroups reflects the actual occupational distribution in public hospitals rather than sampling bias. However, given the significant intergroup differences observed in Table 1 and the theoretical relevance of professional identity in shaping retirement attitudes, the absence of subgroup-specific modeling remains a limitation. We addressed this by providing in-depth descriptive comparisons and integrated interpretation across groups, but future studies with larger stratified samples should adopt profession-specific or interaction-term modeling approaches to yield more nuanced insights. Third, we employed single-item measures for job satisfaction and fatigue to reduce respondent burden in high-pressure clinical settings. While this approach enhanced feasibility, it may have limited the psychometric precision of these constructs. Future research should consider validated multi-item instruments, such as the Maslach Burnout Inventory or the Multidimensional Fatigue Inventory, to capture psychosocial dimensions more comprehensively. Fourth, although we included variables such as education level and job satisfaction as partial proxies for socioeconomic status and intrinsic motivation, we did not directly assess perceived work meaning, financial preparedness, or retirement planning—factors that may influence retirement intention. This omission reflects a practical tradeoff during instrument design, but it limits the conceptual completeness of our analysis. Lastly, although our sample was recruited from healthcare institutions affiliated with a single university system in Sichuan Province, it encompassed a broad range of clinical departments and occupational groups, including doctors, nurses, technicians, and administrative staff. This may still limit the external generalizability of the findings to other regions or healthcare systems with different institutional structures or cultural contexts. However, the study offers timely and theory-informed insights from a middle-income country currently undergoing retirement policy reform. As such, it contributes valuable evidence to inform workforce planning and targeted retention strategies in similar healthcare settings.

In conclusion, this study offers context-specific evidence on the demographic, occupational, and psychosocial factors associated with support for delayed retirement among healthcare professionals in a middle-income setting. By applying Role Theory and the Push-Pull Model as interpretive frameworks, the findings provide a preliminary understanding of how professional identity, job satisfaction, fatigue, and structural characteristics shape retirement attitudes. While the cross-sectional design limits causal inference, the study identifies several practical and theoretically relevant patterns that may inform future profession-specific workforce planning. These findings may serve as a starting point for more in-depth, longitudinal, or intervention-based research aimed at addressing delayed retirement in diverse healthcare contexts.

## Supplementary information

Below is the link to the electronic supplementary material.

ESM 1 (DOCX 17.4 KB)

## Data Availability

The datasets generated and/or analyzed during the current study are available from the corresponding author on reasonable request.

## Abbreviations

CI: Confidence interval
OR: Odds ratio
SD: Standard deviation
SPSS: Statistical Package for the Social Sciences
