# Supplementary material for: Factors influencing healthcare workers’ attitudes toward delayed retirement: a cross-sectional survey
Source: BMC Public Health. 2025 Sep 24;25:3070. doi: 10.1186/s12889-025-24449-7 (PMC12462210; doi:10.1186/s12889-025-24449-7)
Supplement: Supplementary file 1 — (DOCX 17.4 KB) [file 12889_2025_24449_MOESM1_ESM.docx]

**Supplementary File 1: Healthcare Workers' Attitudes Toward Delayed Retirement Survey Questionnaire (English Version)**

**Healthcare Workers' Attitudes Toward Delayed Retirement: Survey Questionnaire**

*Section 1: Demographic Information*

1. **Age**: _______
2. **Gender**:
   - Male
   - Female
   - Prefer not to say
3. **Education Level**:
   - High school or below
   - Bachelor’s degree
   - Master’s degree or higher
4. **Marital Status**:
   - Single
   - Married
   - Divorced
   - Widowed
5. **Years of Work Experience**: ______ years
6. **Professional Group**:
   - Doctor
   - Nurse
   - Technician
   - Administrative Staff

*Section 2: Work-Related Factors*
7. **Weekly Working Hours**:

- Less than 30 hours
- 30-40 hours
- 41-50 hours
- More than 50 hours

1. **Night Shifts Per Month**:
   - None
   - 1-2 shifts
   - 3-5 shifts
   - 6 or more shifts

*Section 3: Job Satisfaction*
9. **Overall, how satisfied are you with your current job?** (5-point Likert scale: 1 = Very dissatisfied, 5 = Very satisfied)

- 1
- 2
- 3
- 4
- 5

*Section 4: Occupational Fatigue*
10. **On a scale of 1 to 10, how fatigued do you feel as a result of your work?**

- (1 = No fatigue, 10 = Extreme fatigue)
- 1 2 3 4 5 6 7 8 9 10

*Section 5: Health Status*
11. **How would you rate your overall health?**

- Excellent
- Good
- Fair
- Poor

1. **Do you have any chronic illnesses (e.g., hypertension, diabetes)?**

- Yes
- No
  If yes, please specify: ____________________

*Section 6: Attitudes Toward Delayed Retirement*
13. **How likely are you to support delayed retirement beyond the traditional retirement age?**

- Very unlikely
- Unlikely
- Neutral
- Likely
- Very likely

1. **Which of the following best describes your view on delayed retirement?**

- I prefer to retire as soon as I am eligible.
- I am open to delaying retirement if it benefits my career/financial situation.
- I strongly support delayed retirement due to personal or professional reasons.
